# The Goldsmith Piano

**Published:** 1890-05

**Authors:** 


### THE GOLDSMITH PIANO.

Those about to supply themselves with a piano, will naturally enquire where they can procure a first-class instrument at the lowest cost.

The Goldsmith Piano and Organ Manufacturing Co., whose advertisement appears in our columns, have made a new departure from old methods. They sell their instruments at their factory, thus dispensing with middle-men, at a saving of nearly one-half to the purchaser. Send for a catalogue.

